# Supplementary material for: A Likelihood Approach for Real-Time Calibration of Stochastic Compartmental Epidemic Models
Source: PLoS Comput Biol. 2017 Jan 17;13(1):e1005257. doi: 10.1371/journal.pcbi.1005257 (PMC5240920; doi:10.1371/journal.pcbi.1005257)
Supplement: S1 File — (TAR.GZ) [file pcbi.1005257.s014.tar.gz › HSPH_Online-SI-Revision/IPoi09/n100-mild/n100-mild_BENCH_table8.pdf]

1	R0	{1.24125, 3.38982}	{1.85533, 9.28761}	{{1.85533, 9.28761}, {1.85533, 9.28761}}	{49.4727, 173.985}	inf	161.	1483.24	{488., 2878.}	821.266	0.864169	newly	287.	930.738	{304., 1830.}	226.058	0.415434	Reff	0.815836	0.483554	{0.459398, 0.509177}	40.7291	ar	34926.	75291.3	{74879., 75728.}	115.574
2	R0	{1.29554, 4.51878}	{2.12117, 12.1677}	{{2.12117, 12.1677}, {2.12117, 12.1677}}	{63.7286, 169.27}	inf	262.	2699.52	{999., 4724.}	930.351	0.899827	newly	367.	1321.08	{447., 2277.}	260.008	0.438708	Reff	0.75267	0.407919	{0.379159, 0.436091}	45.8037	ar	42828.	82651.9	{82375., 82924.}	92.9857
3	R0	{1.2443, 3.64292}	{1.93419, 8.56054}	{{1.93419, 8.56054}, {1.93419, 8.56054}}	{55.444, 134.991}	inf	255.	679.878	{292., 1099.}	166.734	0.37371	newly	387.	447.366	{198., 713.}	44.7407	0.175891	Reff	0.790168	0.438304	{0.429874, 0.44643}	44.5302	ar	37087.	77863.7	{77421., 78275.}	109.949
4	R0	{1.38465, 3.43331}	{2.20704, 7.29612}	{{2.20704, 7.29612}, {2.20704, 7.29612}}	{59.3933, 112.51}	inf	64.	224.909	{79., 485.}	252.33	0.449526	newly	86.	162.873	{52., 351.}	105.074	0.264348	Reff	0.700356	0.343322	{0.336861, 0.349794}	50.9789	ar	49511.	84560.8	{84251., 84856.}	70.7919
5	R0	{1.25409, 4.1068}	{1.93419, 8.56054}	{{1.93419, 8.56054}, {1.93419, 8.56054}}	{54.2306, 108.448}	inf	297.	679.878	{292., 1099.}	130.738	0.31542	newly	386.	447.366	{198., 713.}	44.8755	0.175973	Reff	0.780997	0.438304	{0.429874, 0.44643}	43.8789	ar	38571.	77863.7	{77421., 78275.}	101.871
6	R0	{1.28821, 4.2016}	{2.01028, 10.1103}	{{2.01028, 10.1103}, {2.01028, 10.1103}}	{56.0522, 140.63}	inf	242.	1252.27	{477., 2519.}	417.467	0.621889	newly	315.	713.458	{264., 1438.}	132.526	0.291244	Reff	0.761538	0.419314	{0.403222, 0.43605}	44.9385	ar	41468.	80025.	{79657., 80441.}	92.9812
7	R0	{1.24369, 2.56007}	{1.84836, 6.13642}	{{1.84836, 6.13642}, {1.84836, 6.13642}}	{48.619, 139.697}	inf	25.	220.07	{70., 354.}	780.279	0.776263	newly	53.	189.674	{65., 322.}	260.246	0.400837	Reff	0.764869	0.466319	{0.457931, 0.475916}	39.0328	ar	38603.	74960.1	{74591., 75406.}	94.182
8	R0	{1.232, 3.53942}	{1.93816, 10.0503}	{{1.93816, 10.0503}, {1.93816, 10.0503}}	{57.3182, 183.953}	inf	101.	1344.84	{467., 2318.}	1231.53	0.985722	newly	163.	793.533	{275., 1335.}	386.83	0.54169	Reff	0.809486	0.451742	{0.430775, 0.461689}	44.1939	ar	34555.	77897.5	{77498., 78370.}	125.431
9	R0	{1.36262, 3.85631}	{2.33832, 9.79994}	{{2.33832, 9.79994}, {2.33832, 9.79994}}	{71.6047, 154.127}	inf	139.	1143.31	{388., 2284.}	722.525	0.756205	newly	200.	653.491	{221., 1280.}	229.873	0.368625	Reff	0.708181	0.318976	{0.306671, 0.328651}	54.9584	ar	48410.	86902.8	{86650., 87184.}	79.5141
10	R0	{1.28702, 4.67968}	{1.98131, 11.1772}	{{1.98131, 11.1772}, {1.98131, 11.1772}}	{53.9455, 138.845}	inf	267.	1489.85	{523., 3259.}	458.124	0.63206	newly	358.	791.872	{265., 1711.}	132.426	0.295112	Reff	0.767643	0.435123	{0.416967, 0.458475}	43.317	ar	41092.	79152.9	{78853., 79488.}	92.6236
11	R0	{1.35752, 3.47129}	{2.15962, 10.7214}	{{2.15962, 10.7214}, {2.15962, 10.7214}}	{59.0857, 208.859}	inf	143.	1933.63	{752., 3294.}	1252.19	1.03461	newly	225.	1042.94	{410., 1769.}	363.529	0.566562	Reff	0.755256	0.380144	{0.359749, 0.397996}	49.6669	ar	44669.	83588.6	{83290., 83907.}	87.129
12	R0	{1.35929, 5.83647}	{2.18932, 13.5121}	{{2.18932, 13.5121}, {2.18932, 13.5121}}	{61.0635, 131.512}	inf	598.	3385.24	{1327., 6655.}	466.094	0.663509	newly	613.	1509.2	{594., 2966.}	149.921	0.317066	Reff	0.753917	0.392003	{0.359727, 0.439397}	48.0045	ar	46715.	84192.2	{83771., 84524.}	80.2252
13	R0	{1.28019, 3.29908}	{2.06386, 8.81239}	{{2.06386, 8.81239}, {2.06386, 8.81239}}	{61.2151, 167.117}	inf	135.	719.44	{311., 1212.}	432.919	0.655645	newly	244.	452.2	{194., 759.}	95.2295	0.251842	Reff	0.759998	0.394516	{0.383878, 0.403278}	48.0898	ar	40941.	81347.9	{81053., 81619.}	98.6955
14	R0	{1.36661, 3.48318}	{2.26582, 8.12562}	{{2.26582, 8.12562}, {2.26582, 8.12562}}	{65.7986, 133.282}	inf	77.	388.696	{105., 692.}	404.801	0.535372	newly	115.	256.857	{69., 448.}	141.335	0.273986	Reff	0.705239	0.329725	{0.319526, 0.336701}	53.2464	ar	48672.	85637.7	{85350., 86000.}	75.9487
15	R0	{1.35865, 3.84123}	{2.33968, 11.4691}	{{2.33968, 11.4691}, {2.33968, 11.4691}}	{72.2062, 198.579}	inf	163.	1947.33	{663., 3984.}	1094.68	0.947868	newly	228.	984.345	{315., 2036.}	331.73	0.50316	Reff	0.704243	0.330132	{0.311552, 0.35273}	53.1224	ar	48598.	86868.	{86621., 87099.}	78.7481
16	R0	{1.22879, 3.98329}	{1.80156, 10.0119}	{{1.80156, 10.0119}, {1.80156, 10.0119}}	{46.6125, 151.348}	inf	367.	2061.88	{856., 3423.}	461.82	0.661085	newly	573.	1256.51	{509., 2099.}	127.817	0.285959	Reff	0.822699	0.52696	{0.490673, 0.549206}	37.4973	ar	34405.	73378.6	{72838., 73857.}	113.279
17	R0	{1.34925, 3.64524}	{2.22376, 9.35432}	{{2.22376, 9.35432}, {2.22376, 9.35432}}	{64.8145, 156.617}	inf	162.	1056.82	{400., 1771.}	552.361	0.697533	newly	237.	630.684	{239., 1053.}	168.924	0.321239	Reff	0.721606	0.34908	{0.336499, 0.359449}	51.6246	ar	46871.	84873.7	{84562., 85179.}	81.0794
18	R0	{1.33054, 2.25275}	{2.17292, 6.16453}	{{2.17292, 6.16453}, {2.17292, 6.16453}}	{63.3111, 173.645}	inf	9.	107.472	{41., 204.}	1094.13	1.00276	newly	21.	85.0189	{33., 163.}	305.211	0.530512	Reff	0.740885	0.352747	{0.346646, 0.359162}	52.3884	ar	44336.	83825.5	{83511., 84088.}	89.0688
19	R0	{1.28394, 3.69859}	{2.04871, 11.6322}	{{2.04871, 11.6322}, {2.04871, 11.6322}}	{59.5643, 214.504}	inf	204.	2628.92	{1079., 4418.}	1188.69	1.0291	newly	360.	1350.86	{522., 2292.}	275.24	0.490038	Reff	0.786067	0.429591	{0.402428, 0.459505}	45.3493	ar	39558.	80947.3	{80601., 81193.}	104.629
20	R0	{1.2883, 2.26493}	{1.98552, 5.56151}	{{1.98552, 5.56151}, {1.98552, 5.56151}}	{54.1194, 145.549}	inf	22.	209.043	{50., 555.}	850.193	0.782496	newly	37.	186.638	{39., 471.}	407.648	0.517175	Reff	0.763434	0.415584	{0.408143, 0.425378}	45.5638	ar	40768.	79224.6	{78759., 79585.}	94.3302
21	R0	{1.34098, 2.78621}	{2.17292, 6.16453}	{{2.17292, 6.16453}, {2.17292, 6.16453}}	{62.0397, 121.251}	inf	39.	107.472	{41., 204.}	178.374	0.379965	newly	64.	85.0189	{33., 163.}	61.7925	0.213947	Reff	0.744378	0.352747	{0.346646, 0.359162}	52.6118	ar	44605.	83825.5	{83511., 84088.}	87.9285
22	R0	{1.33783, 5.95911}	{2.11132, 12.644}	{{2.11132, 12.644}, {2.11132, 12.644}}	{57.8168, 112.179}	inf	686.	2636.4	{1128., 5366.}	284.315	0.512422	newly	709.	1244.5	{504., 2579.}	88.0276	0.233367	Reff	0.760757	0.406674	{0.385189, 0.447241}	46.5436	ar	45418.	82482.7	{82204., 82804.}	81.608
23	R0	{1.33359, 3.3069}	{2.16209, 9.852}	{{2.16209, 9.852}, {2.16209, 9.852}}	{62.1255, 197.923}	inf	84.	1450.42	{514., 2437.}	1626.69	1.09557	newly	143.	841.5	{306., 1375.}	488.462	0.623605	Reff	0.719592	0.373926	{0.356183, 0.389436}	48.0364	ar	46245.	83625.9	{83271., 83980.}	80.8324
24	R0	{1.30588, 2.56123}	{2.0598, 5.9936}	{{2.0598, 5.9936}, {2.0598, 5.9936}}	{57.7327, 134.013}	inf	11.	100.68	{29., 206.}	815.273	0.842062	newly	29.	84.02	{27., 180.}	193.448	0.363726	Reff	0.752997	0.386938	{0.380115, 0.39266}	48.6135	ar	42383.	81278.	{80868., 81557.}	91.7702
25	R0	{1.29813, 4.51849}	{2.15363, 13.5067}	{{2.15363, 13.5067}, {2.15363, 13.5067}}	{65.9025, 198.921}	inf	473.	3633.98	{1563., 6117.}	668.283	0.798734	newly	633.	1642.48	{706., 2863.}	160.861	0.32935	Reff	0.756044	0.411502	{0.377273, 0.451724}	45.6457	ar	43371.	83421.9	{83042., 83769.}	92.3448
26	R0	{1.33231, 3.99953}	{2.18735, 12.1087}	{{2.18735, 12.1087}, {2.18735, 12.1087}}	{64.1773, 202.753}	inf	227.	2596.11	{1099., 4470.}	1043.66	0.966773	newly	332.	1270.74	{504., 2165.}	282.753	0.489484	Reff	0.747852	0.380897	{0.355488, 0.4105}	49.0679	ar	44733.	84157.2	{83791., 84464.}	88.1323
27	R0	{1.30555, 4.61445}	{2.15363, 13.5067}	{{2.15363, 13.5067}, {2.15363, 13.5067}}	{64.9596, 192.704}	inf	364.	3633.98	{1563., 6117.}	898.346	0.912493	newly	467.	1642.48	{706., 2863.}	251.709	0.454884	Reff	0.764165	0.411502	{0.377273, 0.451724}	46.1825	ar	42624.	83421.9	{83042., 83769.}	95.7157
28	R0	{1.26819, 5.34969}	{1.87927, 9.84456}	{{1.87927, 9.84456}, {1.87927, 9.84456}}	{48.1852, 84.0211}	inf	578.	1680.42	{599., 3571.}	195.075	0.355546	newly	683.	1008.44	{339., 2197.}	84.2589	0.251387	Reff	0.806442	0.480344	{0.456963, 0.506313}	40.4366	ar	38946.	75982.9	{75584., 76490.}	95.098
29	R0	{1.19925, 3.00394}	{1.75445, 7.55154}	{{1.75445, 7.55154}, {1.75445, 7.55154}}	{46.2956, 151.388}	inf	160.	638.341	{217., 1213.}	299.39	0.465838	newly	328.	477.829	{162., 924.}	83.0532	0.237898	Reff	0.835841	0.511854	{0.497755, 0.524054}	38.7619	ar	31727.	71490.9	{70900., 71883.}	125.332
30	R0	{1.35749, 3.75483}	{2.38758, 11.1331}	{{2.38758, 11.1331}, {2.38758, 11.1331}}	{75.882, 196.501}	inf	77.	1833.09	{548., 3124.}	2280.63	1.23615	newly	120.	950.603	{272., 1596.}	692.17	0.750123	Reff	0.704592	0.316095	{0.298543, 0.326693}	55.1378	ar	48244.	87664.5	{87428., 87887.}	81.7107
31	R0	{1.27131, 4.2161}	{2.0072, 10.6431}	{{2.0072, 10.6431}, {2.0072, 10.6431}}	{57.8844, 152.439}	inf	338.	1993.4	{832., 3402.}	489.765	0.679711	newly	482.	1109.89	{404., 1894.}	135.91	0.293327	Reff	0.797836	0.436754	{0.411817, 0.454591}	45.2577	ar	38299.	79852.6	{79507., 80196.}	108.498
32	R0	{1.31827, 6.18119}	{2.11132, 12.644}	{{2.11132, 12.644}, {2.11132, 12.644}}	{60.1584, 104.556}	inf	827.	2636.4	{1128., 5366.}	218.791	0.43124	newly	844.	1244.5	{504., 2579.}	69.8369	0.213639	Reff	0.757649	0.406674	{0.385189, 0.447241}	46.3243	ar	45262.	82482.7	{82204., 82804.}	82.2339
33	R0	{1.20398, 1.35167}	{1.82289, 4.40955}	{{1.82289, 4.40955}, {1.82289, 4.40955}}	{51.4053, 226.23}	inf	3.	31.7333	{9., 76.}	957.778	0.875564	newly	6.	34.5111	{8., 88.}	475.185	0.610322	Reff	0.802742	0.473291	{0.462814, 0.480805}	41.0407	ar	33329.	74065.1	{73640., 74632.}	122.224
34	R0	{1.32776, 3.146}	{2.13505, 8.31782}	{{2.13505, 8.31782}, {2.13505, 8.31782}}	{60.8009, 164.394}	inf	21.	391.885	{160., 794.}	1766.12	1.18941	newly	25.	256.327	{110., 522.}	925.308	0.93107	Reff	0.733149	0.366169	{0.357407, 0.377221}	50.0553	ar	44872.	83073.6	{82770., 83368.}	85.1346
35	R0	{1.279, 1.11336}	{2.00921, 5.05524}	{{2.00921, 5.05524}, {2.00921, 5.05524}}	{57.0923, 354.053}	inf	0.001	38.7551	{9., 76.}	3.87541×10^{6}	2.42142	newly	0.1	35.2653	{8., 71.}	35165.3	2.37287	Reff	0.754188	0.403749	{0.396196, 0.410542}	46.4658	ar	41033.	79930.1	{79603., 80309.}	94.7947
36	R0	{1.20029, 1.94717}	{1.79887, 4.61852}	{{1.79887, 4.61852}, {1.79887, 4.61852}}	{49.8696, 137.191}	inf	45.	61.3023	{24., 134.}	70.0258	0.231348	newly	117.	63.6977	{23., 140.}	57.1258	0.407099	Reff	0.807351	0.483934	{0.476323, 0.492423}	40.059	ar	33234.	73152.6	{72671., 73553.}	120.114
37	R0	{1.33216, 3.69685}	{2.15962, 10.7214}	{{2.15962, 10.7214}, {2.15962, 10.7214}}	{62.1142, 190.014}	inf	150.	1304.67	{499., 2235.}	769.778	0.837596	newly	219.	703.235	{283., 1194.}	221.81	0.408596	Reff	0.735299	0.371204	{0.356661, 0.383354}	49.5167	ar	45235.	83588.6	{83290., 83907.}	84.7875
38	R0	{1.25434, 2.04314}	{2.02938, 5.7296}	{{2.02938, 5.7296}, {2.02938, 5.7296}}	{61.7887, 180.431}	inf	2.	149.224	{50., 340.}	7361.22	1.76434	newly	6.	129.531	{45., 293.}	2058.84	1.22816	Reff	0.769437	0.399371	{0.391224, 0.407398}	48.0957	ar	38662.	80417.2	{80074., 80858.}	108.001
39	R0	{1.27466, 3.0741}	{1.89934, 6.19362}	{{1.89934, 6.19362}, {1.89934, 6.19362}}	{49.0076, 101.478}	inf	79.	147.833	{39., 307.}	113.11	0.288671	newly	134.	124.024	{32., 247.}	61.9581	0.313375	Reff	0.765178	0.445425	{0.438729, 0.455215}	41.7881	ar	40181.	76667.4	{76233., 77080.}	90.8051
40	R0	{1.33045, 2.68154}	{2.27423, 6.876}	{{2.27423, 6.876}, {2.27423, 6.876}}	{70.9369, 156.42}	inf	10.	177.796	{44., 327.}	1677.96	1.05384	newly	17.	130.815	{33., 241.}	669.499	0.689877	Reff	0.719255	0.325366	{0.320189, 0.330218}	54.7635	ar	45951.	85776.6	{85543., 86036.}	86.6697
41	R0	{1.37003, 2.83984}	{2.33614, 6.89476}	{{2.33614, 6.89476}, {2.33614, 6.89476}}	{70.5174, 142.787}	inf	53.	221.148	{68., 398.}	318.029	0.479488	newly	99.	161.537	{48., 302.}	92.125	0.237095	Reff	0.701168	0.308821	{0.29989, 0.316477}	55.9561	ar	48926.	86878.2	{86567., 87224.}	77.5707
42	R0	{1.3003, 2.94885}	{2.19024, 8.25876}	{{2.19024, 8.25876}, {2.19024, 8.25876}}	{68.4411, 180.067}	inf	49.	495.241	{195., 850.}	910.695	0.923439	newly	90.	322.352	{121., 562.}	258.416	0.472916	Reff	0.726881	0.351514	{0.343364, 0.359681}	51.6407	ar	44237.	84214.3	{83888., 84492.}	90.3708
43	R0	{1.33756, 2.92653}	{2.37035, 7.13655}	{{2.37035, 7.13655}, {2.37035, 7.13655}}	{77.2145, 143.857}	inf	64.	293.474	{88., 584.}	359.649	0.502506	newly	97.	208.719	{67., 394.}	130.657	0.257488	Reff	0.706272	0.301254	{0.294848, 0.310682}	57.3458	ar	47342.	87416.6	{87167., 87654.}	84.6491
44	R0	{1.35801, 2.92276}	{2.14326, 6.48739}	{{2.14326, 6.48739}, {2.14326, 6.48739}}	{57.8236, 121.961}	inf	37.	140.66	{49., 287.}	281.568	0.468043	newly	67.	111.12	{44., 212.}	87.6418	0.224341	Reff	0.707754	0.362307	{0.355438, 0.370034}	48.809	ar	47940.	83177.5	{82750., 83479.}	73.5033
45	R0	{1.31342, 4.02649}	{2.03761, 8.65482}	{{2.03307, 8.59564}, {2.03307, 8.59564}}	{55.1375, 114.947}	inf	207.	810.76	{259., 1434.}	291.671	0.49302	newly	308.	519.92	{170., 935.}	89.7013	0.245955	Reff	0.755532	0.405747	{0.39283, 0.41804}	46.2965	ar	42995.	80640.2	{80124., 80954.}	87.5571
46	R0	{1.37919, 3.75889}	{2.53075, 11.6597}	{{2.53075, 11.6597}, {2.53075, 11.6597}}	{83.4954, 210.19}	inf	174.	2305.81	{983., 4712.}	1225.18	1.01106	newly	255.	1135.37	{495., 2310.}	345.241	0.533849	Reff	0.704463	0.28533	{0.267829, 0.310169}	59.4968	ar	49157.	89653.4	{89455., 89835.}	82.3817
47	R0	{1.37458, 3.56117}	{2.22376, 9.35432}	{{2.22376, 9.35432}, {2.22376, 9.35432}}	{61.7774, 162.675}	inf	54.	1056.82	{400., 1771.}	1857.08	1.17465	newly	75.	630.684	{239., 1053.}	740.912	0.806727	Reff	0.710232	0.34908	{0.336499, 0.359449}	50.8499	ar	48478.	84873.7	{84562., 85179.}	75.0768
48	R0	{1.33393, 3.78296}	{2.21449, 13.2892}	{{2.21449, 13.2892}, {2.21449, 13.2892}}	{66.0125, 251.291}	inf	157.	3460.04	{1380., 6085.}	2103.84	1.27265	newly	214.	1567.39	{614., 2798.}	632.423	0.792712	Reff	0.740251	0.383948	{0.352724, 0.418229}	48.1327	ar	44885.	84687.6	{84379., 85041.}	88.677
49	R0	{1.24918, 2.39046}	{1.84836, 6.13642}	{{1.84836, 6.13642}, {1.84836, 6.13642}}	{47.9659, 156.705}	inf	26.	220.07	{70., 354.}	746.422	0.759229	newly	69.	189.674	{65., 322.}	181.159	0.307713	Reff	0.801174	0.466319	{0.457931, 0.475916}	41.7955	ar	35969.	74960.1	{74591., 75406.}	108.402
50	R0	{1.28281, 2.3032}	{1.98388, 6.25659}	{{1.98388, 6.25659}, {1.98388, 6.25659}}	{54.6511, 171.648}	inf	27.	201.	{49., 601.}	644.444	0.68287	newly	59.	164.395	{40., 477.}	191.407	0.330145	Reff	0.767787	0.414649	{0.404295, 0.423043}	45.9942	ar	40223.	79238.6	{78771., 79670.}	96.9981
